# METTL14-mediated m6A modification of DUSP6 mRNA participating in postoperative cognitive dysfunction due to sevoflurane anesthesia

**DOI:** 10.1016/j.jphyss.2025.100048

**Published:** 2025-10-25

**Authors:** Shengfeng Deng, Guo Mu, Jun Li, Xuan Yu, Qiang Li, Bin Lu

**Affiliations:** Department of Anesthesiology, Zigong Fourth People's Hospital, Zigong, Sichuan Province 643000, PR China

**Keywords:** Sevoflurane, Postoperative cognitive dysfunction, METTL14, DUSP6, m6A methylation

## Abstract

**Background:**

To investigate the mechanisms underlying sevoflurane-induced POCD, C57BL/6 J mice and SH-SY5Y cells were treated with sevoflurane for model establishment.

**Methods:**

After the treatment with sevoflurane, CCK-8, EdU and flow cytometry were employed to detect cell damage. The levels of N6-methyladenosine (m6A), METTL14 and DUSP6 were determined by qPCR and Western blot. The interaction between METTL14 and DUSP6 was analyzed using RIP-qPCR and Me-RIP methodologies. The cognitive function in mice were assessed by water maze test.

**Results:**

After sevoflurane treatment, the cell viability, cell proliferation and METTL14 expression were markedly suppressed, while apoptosis was significantly enhanced. METTL14 overexpression elevated the levels of m6A and DUSP6, increased the binding level of METTL14 to DUSP6 mRNA, reducing damage to cells and cognitive dysfunction of mice. Knockdown of DUSP6 negated the beneficial effects observed with METTL14 overexpression.

**Conclusion:**

Sevoflurane induced POCD by regulating METTL14/DUSP6 through m6A methylation.

## Highlights

• Sevoflurane suppressed cell growth and induced apoptosis in vivo and in vitro.
• Sevoflurane induced postoperative cognitive dysfunction by regulating METTL14/DUSP6 through m6A methylation.
• Overexpression of METTL14 and DUSP6 can alleviate postoperative cognitive dysfunction caused by Sevoflurane.

## Introduction

Postoperative cognitive dysfunction (POCD) denotes a decline in cognitive function that occurs after anesthesia and surgical procedures. The clinical symptoms of POCD are memory loss, information processing capability decline, and disorientation, leading to increased risk of long-term cognitive and mortality, decreased quality of life and other serious clinical consequences [12], [31]. Elderly patients are susceptible to POCD. With the development of population aging, the incidence of POCD is increasing, which bring a heavy burden to society [29].

It is demonstrated that long-term exposure to anesthesia can have irreversible effects on the cognitive function of animals [23]. Sevoflurane (Sevo) is an inhaled anesthetic commonly used in clinical practice, which is safe and effective. However, studies in recent years have found that Sevo anesthesia is associated with cognitive decline, especially in elderly rodents and humans [17]. The treatment of Sevo could increases the incidence of POCD [19]. Moreover, Sevo has been reported to impair m6A-mediated mRNA translation, resulting in impaired fine motor skills and cognitive function. Zhang et al., [28].

N6-methyladenosine (m6A) stands out as the most common mRNA modification found in eukaryotic organisms, playing a crucial role in regulating gene expression and impacting various physiological functions [20]. In the brain, the changes of m6A methylation modification level are closely related to learning, memory and brain injury related diseases [4]. Research indicates that m6A modification is crucial for adult neuron diversification and neuronal capacity regulation, playing a vital role in nervous system development and function. [27]. Methyltransferase 14 (METTL14), an important component of the m6A methyl-translocase complex, has the function of assisting RNA substrate binding. METTL14 is reported to play a significant role in the development of neurodegenerative disorders, including Parkinson’s disease [7]. However, the potential mechanism of METTL14 in POCD has not been studied.

Dual-specificity phosphatase 6 (DUSP6) belongs to the family of bispecific protein phosphatases. Early studies showed that DUSP6 could reduce neurotoxicity and had potential neuroprotective effects [10]. Huang et al. found that DUSP6 protected hippocampal neurons from glutamate-induced apoptosis via the ERK1/2 pathway [8]. And modulation of DUSP6 expression could regulate neuroinflammation in an in vitro model of POCD [5]. Thus, this study established a Sevo-induced POCD model to investigate METTL14's role in POCD and its interaction with m6A modification of DUSP6.

## Materials and methods

### Cell culture and treatment

The human neuroblastoma SH-SY5Y cells were obtained from the Cell Bank of Chinese Academy of Sciences in Shanghai, China). These cells were cultured in DMEM/F12 medium (Gibco, C11330500BT, USA) enriched with 10 % fetal bovine serum (FBS, Gibco, 12657–029-GHS, USA) along with 1 % streptomycin and penicillin (Solarbio, P1400, China). The cells were cultured in a humidified incubator with 5 % carbon dioxide (CO_2_) at 37℃. According to Cheng’s study [3], the cells were exposed to 4.1 % sevoflurane for 6 h to establish the POCD model.

### Cell transfection

The SH-SY5Y cells were plated in 6-well plates and allowed to adhere overnight. Following the manufacturer's instructions, the Lipofectamine 2000 kit (Thermo Fisher, 11668019, USA) was utilized for plasmid transfection. The plasmids were obtained from Sangon (Shanghai, China). And the sequences of plasmids were as follows:

oe-METTL14: 5’-GTGCAGAAGGGACTAGGCAG-3’, oe- Negative Control (OE-NC): 5’-GGGCACCC ACGTAATAGACC-3’.

Sh-DUSP6: 5’-CCGGTCTCTGCAATCTACGTGAAAGCTCGAGCTTTCACGTAG

ATTGCAGAGATTTTTG-3’.

Sh-NC: 5’-CCGGTCTCGGCATGGACGAGCTGTACTCGAGTACAGCTCGTCCATGCCGAGATTTTTG-3’.

### CCK-8

Cell viability of SH-SY5Y cells was assessed using the Cell Counting Kit-8 (CCK-8, Beyotime, C0037, China). Cells were adjusted to a density of 2 × 10⁵ cells/well and plated in 96-well plates, followed by incubation at 37°C with 5 % CO₂. After 24 h, the cells were treated with Sevo or transfected. Next, 100 μL/well of 10 % CCK-8 solution was added, and the cells were incubated for 4 h. Absorbance at 450 nm was measured using a microplate reader (Bio-Rad, USA) to determine cell viability.

### EdU

SH-SY5Y cells were plated at 3 × 10⁵ cells/well in 6-well plates and incubated for 24 h. Following drug treatment or transfection, an EdU working solution (10 μM, Beyotime, C0075L, China) was introduced for 2 h. The cells were fixed with 4 % paraformaldehyde for 15 min and subsequently permeabilized using 0.3 % Triton X-100 for 10–15 min at room temperature. Hoechst33342 was employed for cell nuclei staining. Finally, the positive cells were observed with fluorescence microscope (Leica, Germany) and quantification was performed using ImageJ software.

### Cell apoptosis detected by flow cytometry

After drug treatment or transfection, the cells were harvested using trypsin without EDTA (Solarbio, T8150, China). According to the protocol provided with the Annexin V-FITC/PI Apoptosis Detection Kit (KeyGEN, KGA1102, China), approximately 5 × 10⁵ cells were rinsed with PBS and resuspended in 500 μL of Binding Buffer. Then the cells were incubated with Annexin V-FITC and Propidium Iodide for 10 min at room temperature in the dark. The apoptosis rate was detected by flow cytometry (Beckman, BB36862, China).

### Western blot

Total proteins from SH-SY5Y cells and hippocampal tissues of mice were extracted using RIPA buffer (Solarbio, R0010, China). Protein concentration was measured with a BCA kit (Beyotime, P0012S, China), and proteins were separated by SDS-PAGE. The extracted proteins were transferred to PVDF membranes (Servicebio, G6044, China), which were then blocked before being incubated overnight at 4°C with primary antibodies. The antibodies such as Bax (CST, #2772, 1:1000), Bcl-2 (CST, #3498, 1:1000), Caspase-3 (CST, #9662, 1:1000), METTL14 (CST, #51104, 1:1000), DUSP6 (CST, #50945, 1:1000) and β-actin (CST, #4967, 1:1000) were included. After thorough washing with TBST, the membranes were incubated with HRP- conjugated secondary antibody (CST, #7074, 1:2000) for 1 h. Protein bands were visualized using ECL kits (YEASEN, 36208ES76, China), and gray values were analyzed with ImageJ software.

### qPCR

Total RNA was isolated using Trizol (Vazyme, R411, China) and reverse transcribed into cDNA with a cDNA synthesis kit (Vazyme, R312, China). qPCR was performed with the SYBR Green Mix kit (Vazyme, Q311, China), and relative mRNA expression levels of target genes were calculated via the 2^–ΔΔCT^ method. Primer sequences were listed below.

*YTHDF1*: F: 5’- ACCTGTCCAGCTATTACCCG-3’.

R: 5’- TGGTGAGGTATGGAATCGGAG-3’.

*IGF2BP2* F: 5’- AGTGGAATTGCATGGGAAAATCA-3’.

R: 5’- CAACGGCGGTTTCTGTGTC-3’.

*METTL14* F: 5’- GTTGGAACATGGATAGCCGC-3’.

R: 5’- CAATGCTGTCGGCACTTTCA-3’.

*DUSP6* F: 5’- GAAATGGCGATCAGCAAGACG-3’.

R: 5’- CGACGACTCGTATAGC TCCTG-3’.

*GAPDH* F: 5’- GGCACAGTCAAGGCTGAGAATG-3’.

R: 5’- ATGGTGGTGAAGACGCCAGTAC-3’.

### Detection of m6A methylation levels

EpiQuik a m6A RNA Methylation Quantification Kit (AmyJet Scientific, P-9005, China) was employed to detect the levels of m6A methylation. All procedures were performed according to the manufacturer's instructions.

### RNA immunoprecipitation-qPCR assay (RIP- qPCR)

The Magna RIP kit (Millipore, 17–700, USA) was used to verify the interaction between METTL14 and DUSP6. Cells were lysed, and the extracted proteins were incubated with IgG (CST, #3423, 1: 20) or DUSP6 (CST, #50945, 1: 200) antibodies at 4°C for 1 h. Subsequently, RNA was isolated to measure the mRNA expression of *DUSP6* by qPCR examination.

### m6A RNA methylation immunoprecipitation-qPCR assay (MeRIP- qPCR)

The m6A methylation levels of DUSP6 were assessed utilizing the Magna MeRIP m6A Kit from Merck, Germany. Following the protocol, RNA fragments were isolated and mixed with Magnetic beads that were pre-coated with anti-m6A antibodies. After a series of washes with m6A Salt, the bound RNA was recovered using an RNA purification kit from Thermo Fisher, USA, and subsequently analyzed via qPCR.

### RNA stability assay

SH-SY5Y cells were treated with 1 μg/μL actinomycin D and collected for 0, 8, 16 and 24 h respectively. Total RNA was subsequently isolated, and qPCR was performed to measure RNA levels at each time point. The stability curve was obtained. A more gradual curve indicates a slower degradation rate and consequently, higher stability.

### Animal POCD model

To avoid potential effects of estrus-cycle-related hormonal fluctuations on cognitive tests [18], male C57BL/6 J mice, aged 6–8 weeks, were obtained from SJA Laboratory Animal Co., Ltd in Hunan, China. These mice were divided randomly into three groups, each containing six mice: Control, Sevo+AAV-oe-NC and Sevo+AAV-oe-METTL14 group. According to previous experiment [25], all mice except those in the control group were subjected to 4 % Sevo in pure oxygen for six hours. The control group received only fresh oxygen. In addition, the Sevo+AAV-oe-NC and Sevo+AAV-oe-METTL14 groups were injected with adeno-associated virus (AAV) oe-NC and AAV-oe-METTL14 (Genechem, China) respectively through tail vein 2 w before Sevo anesthesia. In order to cross the blood–brain barrier efficiently, the AAV serotype was PHP.B.

### Morris water maze test

24 h after Sevo exposure, the cognitive abilities of the mice were tested using the Morris water maze. As described previously [16], the mice were trained to reach the underwater platform for 5 consecutive days. Subsequently, the platform was removed and the mice were released to water for 60 s. The swimming path, escape latency and duration in the target quadrant were documented.

### Statistical analysis

The results were presented as mean ± SD and statistical analysis was performed using GraphPad Prism 9 software. All quantitative data presented as relative values were normalized such that the mean of the respective control group was set to 1. Comparisons between two groups were conducted using an unpaired *t*-test. ANOVA was used for comparisons involving multiple groups, followed by the Tukey's HSD test for post hoc comparisons. A p-value of less than 0.05 was deemed to indicate statistical significance*.*

## Results

### Sevoflurane stimulation induced damage to SH-SY5Y cells and inhibited the expression of METTL14

**Fig. 1:**
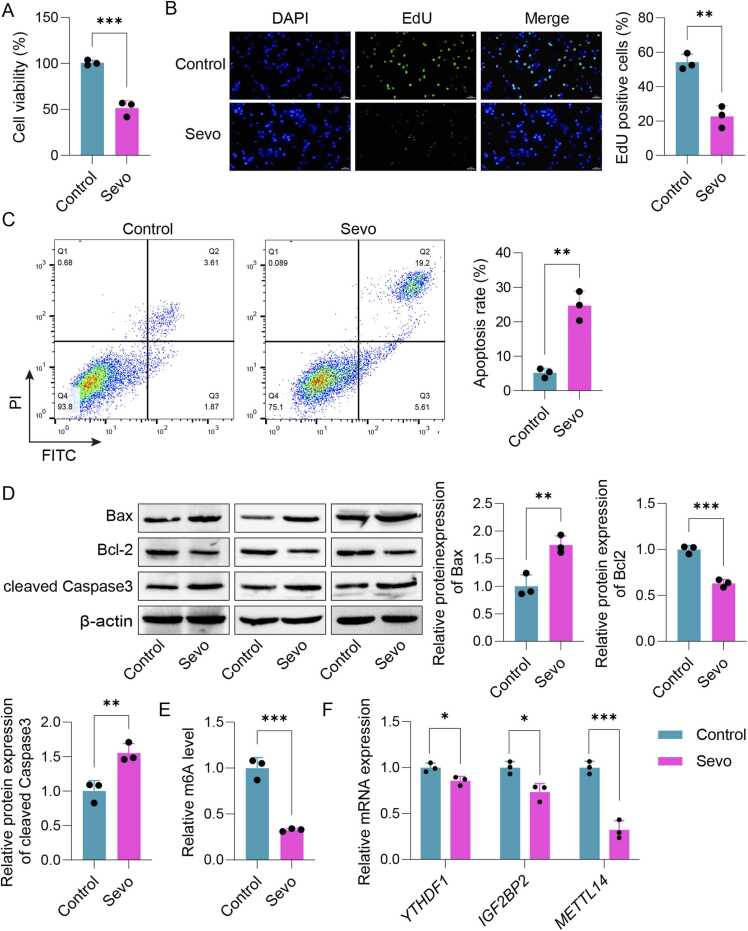
Effects of Sevo on SH-SY5Y cell damage and METTL14 expression. (A) The cell viability was measured by CCK-8. (B) The cell proliferation was detected using EdU assay. (C) Cell apoptosis was detected by flow cytometry. (D) The protein expressions of Bax, Bcl-2 and caspase-3 in SH-SY5Y cells were determined with Western blot. (E) The m6A levels of SH-SY5Y cells. (F) The mRNA expressions of *YTHDF1*, *IGF2BP2* and *METTL14* were measured using qPCR. n = 3, *p < 0.05, **p < 0.01, ***p < 0.001.

In order to construct POCD cell model, SH-SY5Y cells were subjected to 4.1 % Sevo for 6 h. As depicted in Fig. 1A,B, cell viability and proliferation in Sevo group were markedly suppressed compared to the control group. Additionally, the apoptosis rate was significantly higher in POCD cell model (Fig. 1C). Western Blot analysis indicated a notable increase in the expression levels of Bax and caspase-3, while a significant decrease in Bcl-2 levels was also observed (Fig. 1D). The results above indicated that the POCD cell model were established successfully. Finally, the mRNA expressions of *m6A* and m6A methylation-related genes (*YTHDF1*, *IGF2BP2* and *METTL14*) were detected by RT-PCR. The results showed a marked reduction in m6A levels within SH-SY5Y cells, along with a significant downregulation of YTHDF1, IGF2BP2, and METTL14, with METTL14 exhibiting the lowest level (Fig. 1E,F). Taken together, we concluded that Sevo stimulation could induce injury of SH-SY5Y cells, which might be related to the inhibition of METTL14 expression.

### Overexpression of METTL14 attenuated SH-SY5Y cell injury caused by Sevo

**Fig. 2:**
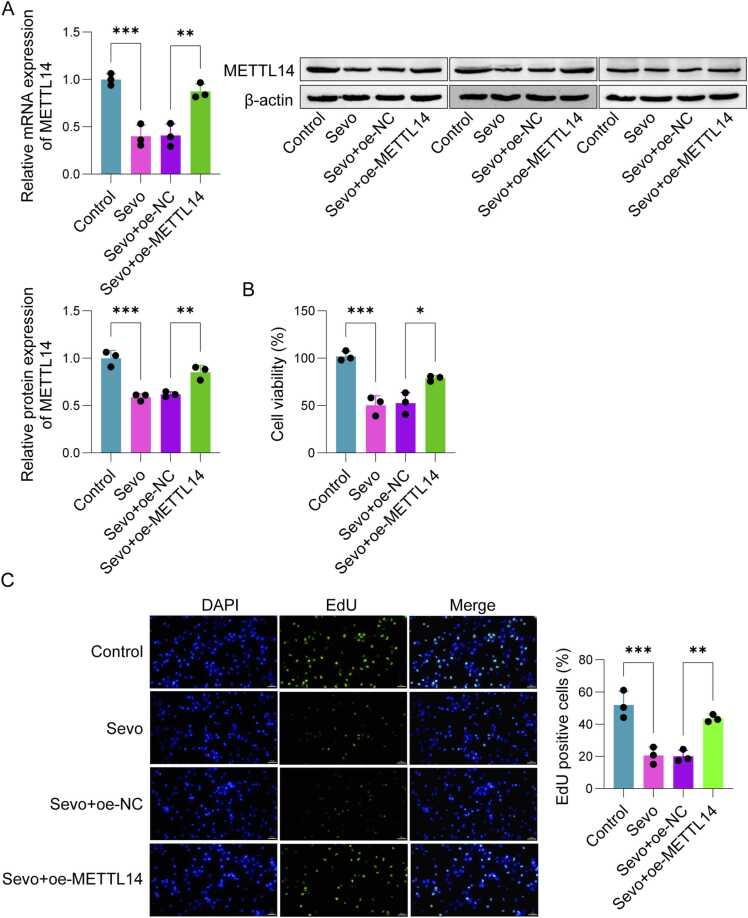

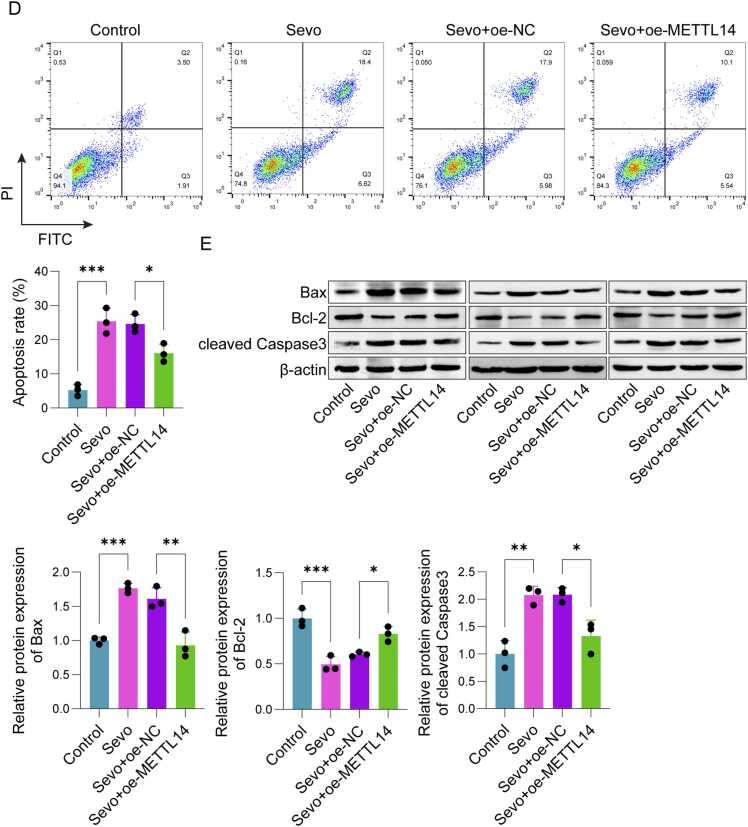
Overexpression of METTL14 alleviate SH-SY5Y cell damage caused by Sevo. The SH-SY5Y cells were transfected with oe-NC or oe-METTL14. (A) qPCR and Western blot were applied to detect the expression of METTL14. (B) CCK-8 was used to detect cell viability. (C) EdU was employed to detect cell proliferation. (D) Cell apoptosis was examined using flow cytometry. (E) Western blot was used to detect the expressions of Bax, Bcl-2 and caspase-3 in SH-SY5Y cells. n = 3, *p < 0.05, **p < 0.01, ***p < 0.001.

After transfected with oe-METTL14, both mRNA and protein levels of METTL14 in SH-SY5Y cells were notably upregulated (Fig. 2A). Overexpression of METTL14 also led to a significant increase in cell viability (Fig. 2B) and proliferation (Fig. 2C), while reducing the apoptosis rate (Fig. 2D). In Sevo-treated SH-SY5Y cells, METTL14 overexpression resulted in a marked decrease in Bax and Caspase-3 protein levels and a significant rise in Bcl-2 levels (Fig. 2E). These results indicated that METTL14 protects SH-SY5Y cells from Sevo-induced damage and may play a crucial role in POCD progression.

### METTL14 regulated the DUSP6 levels via m6A modification

**Fig. 3:**
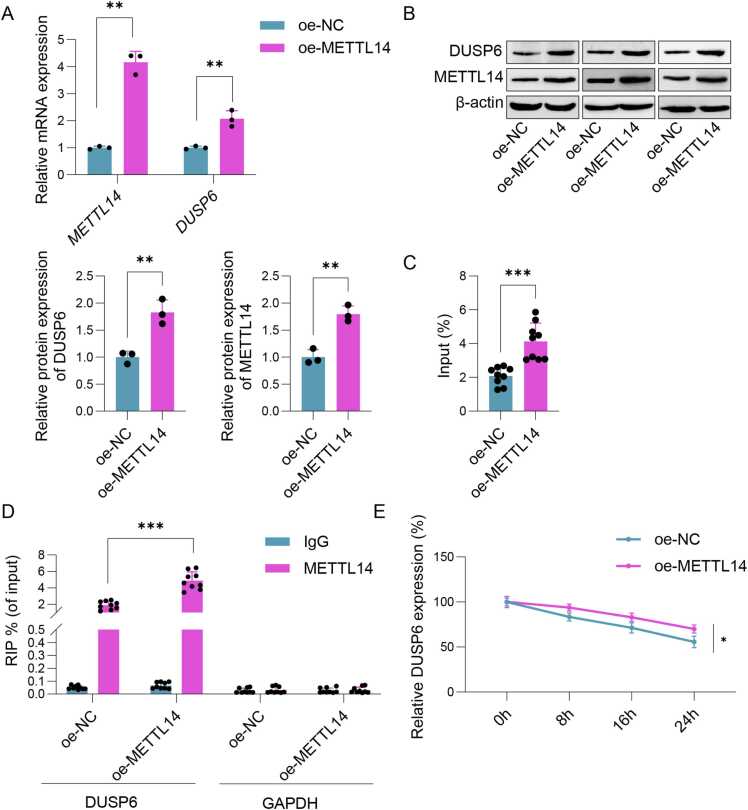
METTL14 modulated the levels of DUSP6 in SH-SY5Y cells. (A) qPCR was applied to detect the mRNA expression of *METTL14* and *DUSP6*. (B) Western blot was applied to detect the protein expression of METTL14 and DUSP6. (C) The m6A level of DUSP6 were detected by Me-RIP. (D) The binding relationship between METTL14 and DUSP6 mRNA was detected by RIP-qPCR. (E) The stability of *DUSP6* mRNA was detected by actinomycin D. n = 3, *p < 0.05, **p < 0.01, ***p < 0.001.

To investigate whether METTL14 can participate in POCD progression through regulating the expression of DUSP6, SH-SY5Y cells were transfected with oe-METTL14. As depicted in Fig. 3A-B, METTL14 overexpression significantly increased the mRNA and protein levels of METTL14 as well as DUSP6. Bioinformatics analysis revealed multiple methylation binding sites for DUSP6 (Fig S1A). Besides, the m6A levels of DUSP6 were notably elevated following METTL14 overexpression (Fig. 3C). Results from RIP-qPCR indicated a notable rise in the interaction between METTL14 and DUSP6 mRNA subsequent to METTL14 overexpression (Fig. 3D). And the stability of DUSP6 is significantly enhanced with the overexpression of METTL14 (Fig. 3E).

### Sevo induced damage to SH-SY5Y cells by regulating METTL14/DUSP6 through m6A methylation

**Fig. 4:**
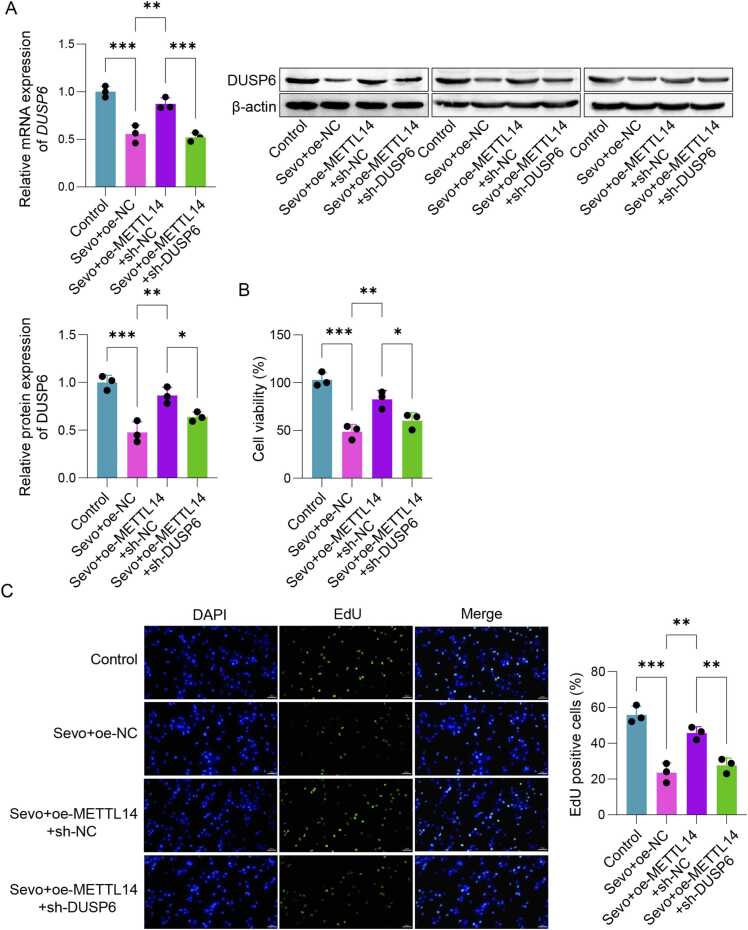

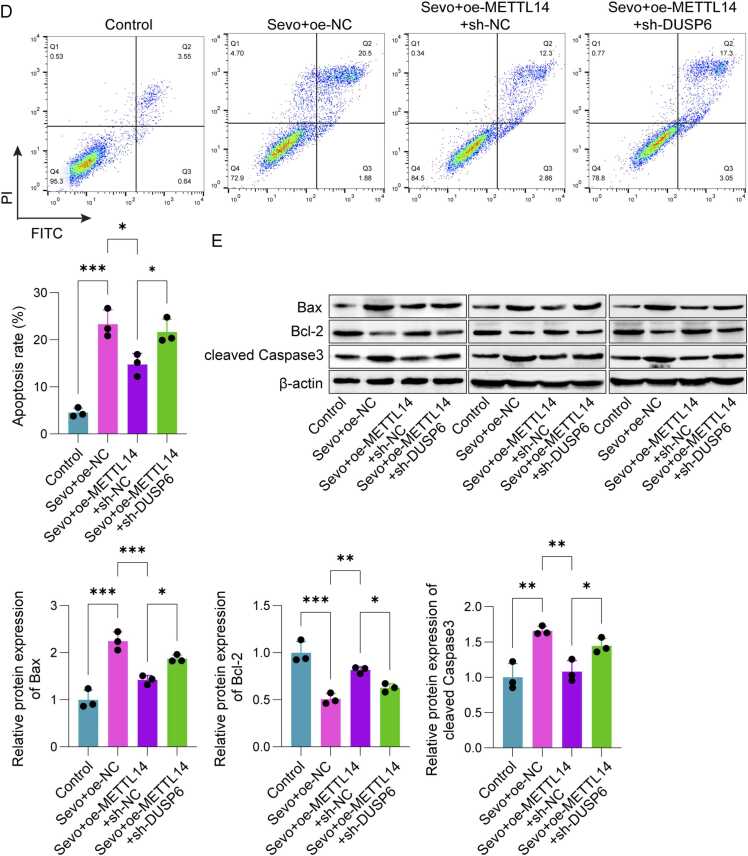
Sevo induced injury in SH-SY5Y cells by regulating METTL14/DUSP6 through m6A methylation. The SH-SY5Y cells were transfected with oe-METTL14 and sh-DUSP6. (A) qPCR and Western blot were applied to detect the expression of DUSP6. (B) CCK-8 was used to detect cell viability. (C) EdU was employed to detect cell proliferation. (D) Cell apoptosis was examined using flow cytometry. (E) Western blot was used to detect the expressions of Bax, Bcl-2 and caspase-3 in SH-SY5Y cells. n = 3, *p < 0.05, **p < 0.01, ***p < 0.001.

As depicted in Fig. 4A, treatment with Sevo led to a decrease in the mRNA expression of *DUSP6* when compared to the control group. However, DUSP6 levels were significantly upregulated following oe-METTL14 transfection and downregulated after sh-DUSP6 transfection. Sevo treatment reduced the mRNA expression of *DUSP6* compared to the control group. However, DUSP6 levels were significantly upregulated following oe-METTL14 transfection and downregulated after sh-DUSP6 transfection. Besides, the overexpression of METTL14 substantially enhanced both cell viability and proliferation while concurrently suppressing cell apoptosis. After sh-DUSP6 treatment, there was a marked inhibition of cell viability and proliferation, alongside a significant rise in the apoptosis rate (Fig. 4B-D). Finally, Western Blot results revealed that Sevo and sh-DUSP6 treatment significantly elevated Bax and Caspase-3 protein levels while reduced Bcl-2 levels in SH-SY5Y cells. Conversely, METTL14 overexpression in Sevo-treated cells markedly decreased Bax and Caspase-3 levels and increased Bcl-2 levels (Fig. 4E). The above results indicated that Sevo regulated METTL14/DUSP6 to induce SH-SY5Y cell damage through m6A methylation, indicating METTL14's role in POCD progression through DUSP6 regulation.

### Sevo induced cognitive dysfunction in mice by regulating METTL14/DUSP6 through m6A methylation

**Fig. 5:**
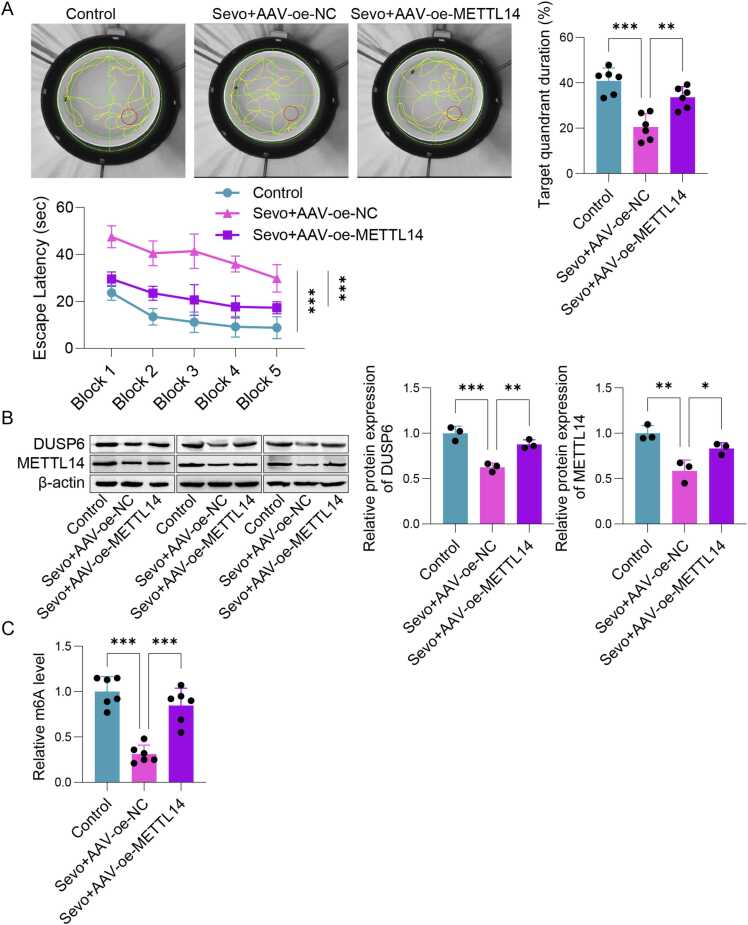
Sevo induced cognitive dysfunction in mice by regulating METTL14/DUSP6 through m6A methylation. The mice were treated with AAV-oe-NC or AAV-oe-METTL14. (A) The mice were subjected to a water maze test. (B) Western blot was employed to detect the expressions of METTL14 and DUSP6 in the hippocampus of POCD mice. (C) The m6A levels of hippocampal tissues in POCD mice. n = 6, *p < 0.05, **p < 0.01, ***p < 0.001.

To further validate the impact and mechanism of Sevo on cognitive dysfunction, an animal model was established. The water maze experiment shown in Fig. 5A was conducted to observe the cognitive function of mice. When compared to the control group, the Sevo+AAV-oe-NC group demonstrated significantly longer escape latencies and reduced time spent in the target quadrant. In contrast, the Sevo+AAV-oe-METTL14 group exhibited a shorter escape latency and a longer duration in the target quadrant relative to the Sevo+AAV-oe-NC group. Besides, levels of METTL14, DUSP6, and m6A in hippocampal tissues were significantly lower in the Sevo+AAV-oe-NC group than those in the control group, whereas they showed an increase in the Sevo+AAV-oe-METTL14 group (Fig. 5B-C). These findings indicated that Sevo induces cognitive dysfunction in mice by regulating METTL14/DUSP6 through m6A methylation.

## Discussion

Sevoflurane, an inhaled anesthetic, is reported to cause neuronal apoptosis and brain tissue damage, which is a key trigger for the increase in the incidence of POCD [19], [22]. m6A methylation plays a vital role in regulating gene expression and various cellular activities, with its significance in the nervous system garnering considerable attention [9]. However, as an m6A methyltransferase, the role and mechanism of METTL14 in POCD induced by Sevo anesthesia remains unclear. This study demonstrated that METTL14 modulates DUSP6 expression via m6A methylation, contributing to neuronal apoptosis and POCD development. Apoptosis, as a programmed cell death mode, is involved in embryonic development and tissue homeostasis maintenance under normal physiological conditions. However, under pathological conditions, excessive apoptosis in the nervous system will destroy the integrity of neural circuits, leading to cognitive impairment [30]. Studies have reported that Sevo could promote neuronal apoptosis in POCD animal models [11]. Our findings revealed a significant rise in the apoptosis rate following Sevo treatment, confirming the POCD cell model induced by Sevo was successfully constructed. m6A methylation, one of the most prevalent mRNA modifications in mammals, is crucial for maintaining central nervous system function. For example, it participates in the control of neural development, regeneration, synaptic formation, and the advancement of neurodegenerative disorders [14], [2], [21], [26]. The methyltransferase METTL14, which is critical for m6A modification, has been demonstrated to be significantly involved in neurological disorders [13]. In Feng et al.’s study, the increased expression of METTL14 could improve neurological deficits and memory impairments, highlighting its significance in traumatic brain injury through m6A methylation [6]. Xu et al. observed the aberrant METTL14 levels in the hippocampus of mice with cognitive dysfunction, while the overexpression of METTL14 could alleviate the impaired cognitive behaviors and enhance m6A level [24]. In the present study, we observed that m6A levels induced by Sevo were notably diminished in SH-SY5Y cells, alongside a reduced expression of METTL14. With the overexpression of METTL14, cell apoptosis induced by Sevo was inhibited in SH-SY5Y cells. In conclusion, the significant role of METTL14 in POCD progression was preliminarily confirmed.

DUSP6 as a highly conserved gene is widely expressed in various human tissues. It is demonstrated that DUSP6 can specifically dephosphorylate and inactivate ERK1/2 in the MAPK family, thereby regulating cell proliferation, apoptosis and other life activities [1]. In the nervous system, DUSP6 is involved in key processes such as neuronal signaling, synaptic plasticity maintenance and the stability of neural circuits through the regulation of ERK1/2, which is closely linked to cognitive function [10]. Examination of brain samples from patients with Alzheimer's disease indicated a substantial decrease in DUSP6 expression within the hippocampus and cortex compared to healthy individuals, accompanied by abnormal increase of ERK1/2 phosphorylation, neuronal apoptosis and so on [15]. By constructing a POCD cell model, Ding et al. confirmed that Sevo could induce neuroinflammation by inhibiting the expression of DUSP6. Whereas, DUSP6 overexpression alleviated the neuroinflammation significantly through ERK1/2 pathway regulation [5]. Our experimental results showed that Sevo treatment significantly reduced DUSP6 expression in SH-SY5Y cells, implying that DUSP6 depletion was the potential causative factors for POCD. In addition, previous research has shown that DUSP6 could be m6A modified by YTHDF1 in POCD [5]. Interestingly, we found that there were m6A modification sites with high feasibility on DUSP6. Therefore, we further verified the interaction between m6A modification of DUSP6 and METTL14. These results illustrated that METTL14 is responsible for the modulation of both m6A and mRNA levels of DUSP6 in SH-SY5Y cells as well as in the brain tissues of mice. Additionally, the silencing of DUSP6 mitigated the effects of METTL14 overexpression on neuronal cell proliferation and apoptosis. In summary, this study revealed that the cognitive impairment caused by Sevo is related to regulation of METTL14/DUSP6 through m6A methylation. This discovery offers novel targets and insights for POCD prevention and treatment. However, we only examined the effects of METTL14 over-expression and DUSP6 knockdown under Sevo exposure. And the influence of these manipulations on basal cell viability, apoptosis, proliferation and cognitive performance in untreated animals or cells was not assessed. Consequently, we cannot exclude the possibility that METTL14 or DUSP6 modulates these parameters independent of Sevo exposure. In future studies, we will conduct multidimensional control experiments to systematically analyze the differential regulatory roles of these two key factors under both physiological and pathological conditions.

## Author contributions

Shengfeng Deng, Guo Mu guaranteed the integrity of the entire study. Shengfeng Deng, Guo Mu, Xuan Yu designed the study and literature research. Shengfeng Deng, Xuan Yu defined the intellectual content. Jun Li, Qiang Li performed experiment. Jun Li, Qiang Li, Bin Lu collected the data. Jun Li, Qiang Li, Bin Lu analyzed the data. Shengfeng Deng and Guo Mu wrote the main manuscript and prepared figures. All authors reviewed the manuscript.

## CRediT authorship contribution statement

**Bin Lu:** Writing – review & editing, Formal analysis, Data curation. **Qiang Li:** Writing – review & editing, Visualization, Project administration, Formal analysis, Data curation. **Xuan Yu:** Writing – review & editing, Conceptualization. **Jun Li:** Writing – review & editing, Visualization, Project administration, Formal analysis, Data curation. **Guo Mu:** Writing – review & editing, Writing – original draft, Supervision, Conceptualization. **Shengfeng Deng:** Writing – review & editing, Writing – original draft, Supervision, Conceptualization.

## Ethics approval and consent to participate

The animal experiments were approved by the Laboratory Animal Welfare and Ethics Committee of Zigong Fourth People's Hospital (2025–002).

## Consent for publication

Not Applicable

## Code availability

Not applicable

## Declaration of Generative AI and AI-assisted technologies in the writing process

During the preparation of this work the authors did not use any AI-assisted technology.

## Funding

Not applicable. This study did not receive any funding in any form.

## Declaration of Competing Interest

The authors declare that they have no known competing financial interests or personal relationships that could have appeared to influence the work reported in this paper.

## Appendix A Supplementary material

Figure S1 The m6(A) methylation sites of DUSP6 predicted by SRAMP database

## Footnotes

Appendix A Supplementary data associated with this article can be found in the online version at doi:10.1016/j.jphyss.2025.100048.
