# Supplementary figures and images for: METTL14-mediated m6A modification of DUSP6 mRNA participating in postoperative cognitive dysfunction due to sevoflurane anesthesia
Source: J Physiol Sci. 2025 Oct 25;75(3):100048. doi: 10.1016/j.jphyss.2025.100048 (PMC12617634; doi:10.1016/j.jphyss.2025.100048)

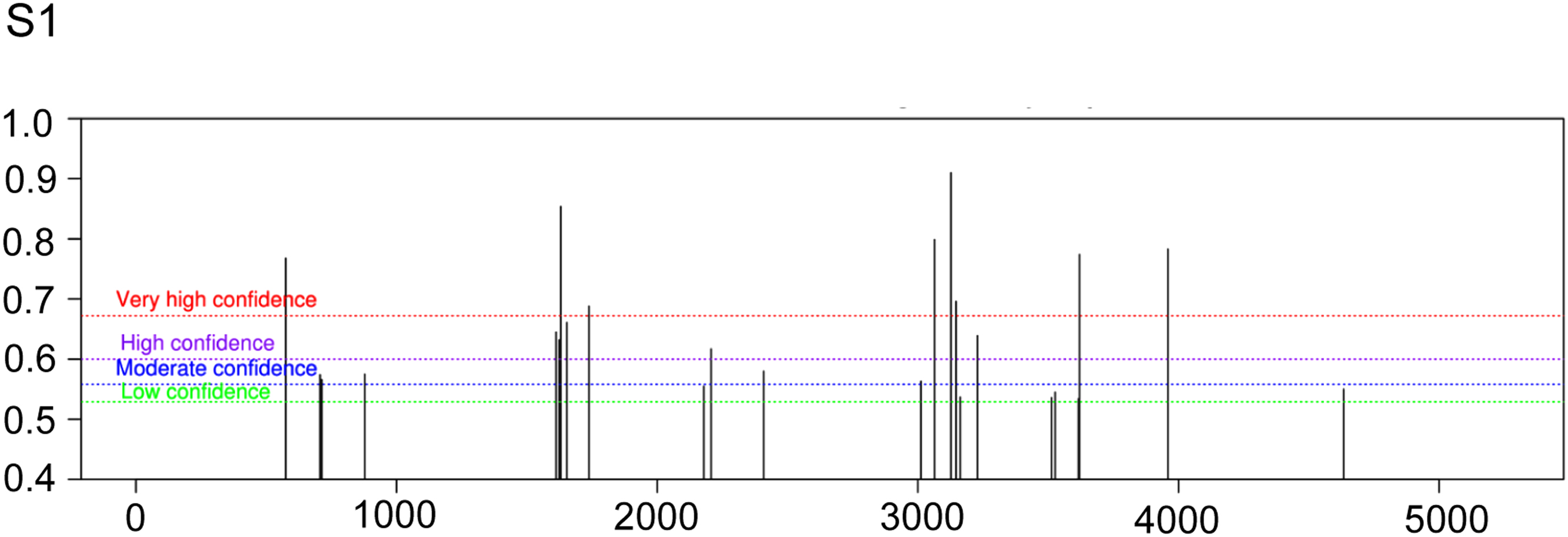

Supplement: Supplementary file 1 — Figure S1 The m6(A) methylation sites of DUSP6 predicted by SRAMP database [file mmc1.jpg]
